# A Case of Hemorrhagic Shock From Newly Diagnosed Cervical Cancer

**DOI:** 10.7759/cureus.81793

**Published:** 2025-04-06

**Authors:** Katherine Griesmer, Landry Hadderton, Luke R Bishop, Derek A Robinett

**Affiliations:** 1 Emergency Medicine, University of Alabama at Birmingham School of Medicine, Birmingham, USA

**Keywords:** arterial embolization, bladder hemorrhage, cervical cancer, hemorrhagic shock, pelvic ultrasound, ultrasound

## Abstract

Cervical cancer remains a prevalent, though preventable, disease with proper screening and vaccination. While the disease is often asymptomatic in the initial stages, severe delayed presentations may include massive hemorrhage. We present a case on newly diagnosed cervical cancer found on evaluation for recurrent urinary tract infections (UTIs) and vaginal hemorrhage requiring arterial embolization for hemorrhagic shock.

## Introduction

Cervical cancer remains one of the leading malignancies in women worldwide, behind only breast and colorectal cancer, with a significant burden, particularly in the developing world [1,2]. In 2018, there were an estimated 13,000 new cases with 4,100 deaths attributed to cervical cancer worldwide [1]. Despite preventative mechanisms, such as human papillomavirus (HPV) vaccination and screening guidelines, it remains a potentially deadly cancer, particularly if discovered in later stages [2]. Given an often early asymptomatic stage, delayed presentations could vary from intermittent vaginal bleeding, particularly post-coital, pelvic or low back pain to hematuria, hematochezia, brisk vaginal bleeding, or evidence of a fistulous tract with the vaginal canal [1]. This case report discusses a newly diagnosed cervical cancer with a presentation consisting of hemorrhagic shock from metastatic invasion into the right uterine artery with a fistulous tract to the bladder. This highlights the need for continued high suspicion for malignant gynecologic presentations despite an increased focus on preventative medicine.

## Case presentation

A 62-year-old female presented to the emergency department with concern for persistent urinary tract infection. Upon arrival to the ED, the patient was noted to also have significant vaginal bleeding with acute onset. Initial exam was notable for tachycardia to 106, hypotension to 94/60 mmHg with significant pulsatile blood and clots noted in the vaginal vault and coming from the urethra, with continued bleeding following Foley catheter placement.

**Figure 1:**
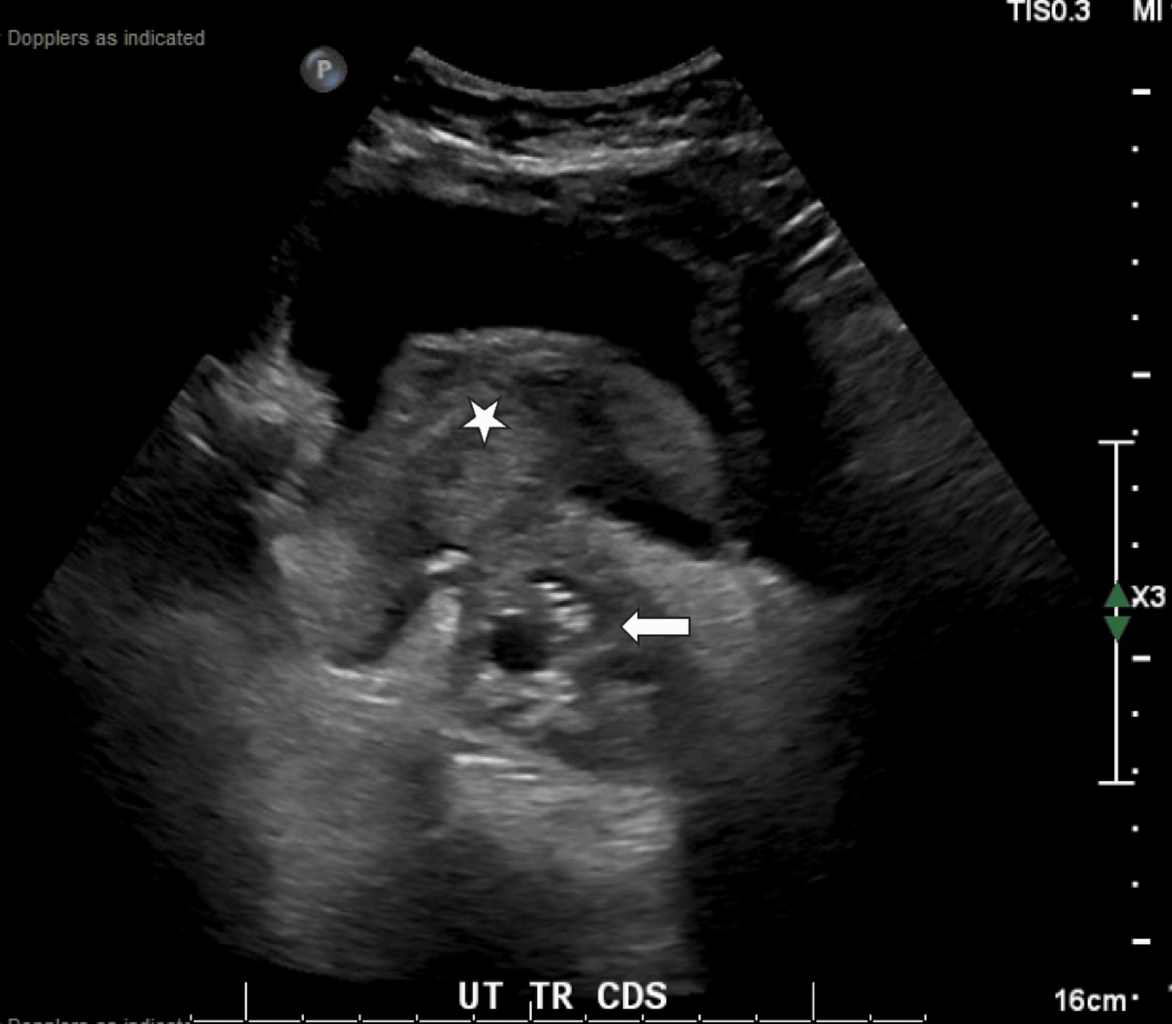
Large cervical mass with protrusion (white arrow) and clot burden in the bladder (white star) on a transvaginal ultrasound in the transverse plane

**Figure 2:**
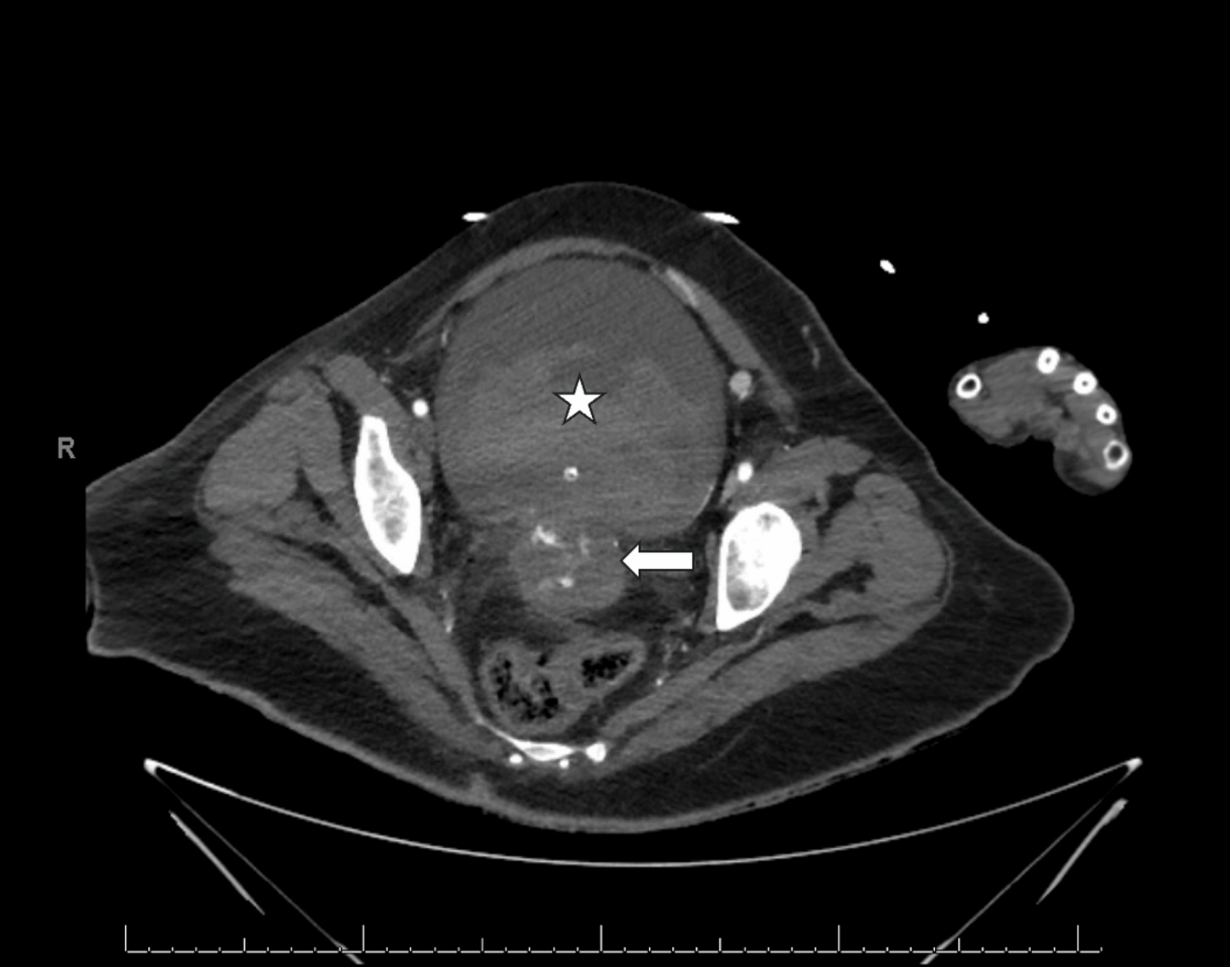
Large cervical mass with active extravasation (white arrow) and fistulous tract with bladder containing a large clot burden (white star) and Foley catheter on CT abdomen and pelvis with contrast axial view

A transvaginal ultrasound was performed with concern for evidence of a mass involving the bladder and uterus with bladder hematoma evident (Figure 1). Vaginal bleeding was not responsive to tranexamic acid (TXA) or vaginal packing. The patient was subsequently transferred from a freestanding ED to a level 1 academic ED. A contrasted computed topography abdomen and pelvis (CTAP) was obtained immediately following transfer, which noted necrotic cervical mass with fistula to the bladder, active extravasation from right uterine artery into the uterus and bladder (Figure 2), obstructive moderate right hydroureteronephrosis secondary to cervical mass, as well as incidental findings of cirrhosis, portal hypertension, and mild interstitial edematous pancreatitis.

Gynecology was consulted and evaluated the patient in the ED. A repeat bedside ultrasound was concerning for abdominal free fluid, a large pelvic mass, and bladder hematoma. Given concern for active bleeding from the right uterine artery, the patient was subsequently taken to interventional radiology with successful embolization. Prior to the procedure, the patient received a total of four liters of crystalloid, five units of packed red blood cells, two units of fresh frozen plasma, and one unit of platelets with blood pressure responsive to interventions. Initial hemoglobin was 7.1 gm/dL on arrival. The patient was also empirically covered with broad-spectrum antibiotics in the setting of leukocytosis of 17,940 cells/µL and concern for possible sepsis from a urinary source contributing to presentation as well.

The patient's course was complicated by obstructive hydronephrosis treated with bilateral nephrostomy tube placement, aspiration pneumonitis, left ovarian vein thrombus, and pulmonary thromboembolism with IVC filter placement, candidemia from Candida albicans, acute right cerebellar and subacute pontine strokes, and a new diagnosis of diabetes mellitus with an initial blood glucose of 644 mg/dL without evidence of diabetic ketoacidosis. She was ultimately diagnosed with Stage IV squamous cell carcinoma of the cervix. She was also found to have a rectovaginal fistula; however, the patient declined ostomy during hospitalization. Given multiple comorbidities, a complicated hospital course, and extensive conversation with radiation oncology, the patient started a course of palliative radiation.

## Discussion

While cervical cancer unfortunately remains a common diagnosis worldwide, with roughly two-thirds of patients presenting with advanced disease, this case highlights the need for further investigation of underlying malignancy, particularly in the setting of vaginal and urethral bleeding [3]. Cervicovaginal bleeding, though rarer, remains a significant complication with a previous focus on ligation of the hypogastric artery. This was unfortunately complicated by frequent recurrence of revascularization hemorrhage [3]. Later interventions focused on arterial embolization due to the direct involvement of the bleeding vessel, limited exposure to anesthesia, and intraoperative complications. Successful embolization occurred in 95.7% within the first 24 hours of presentation in one study involving 47 patients [3]. However, the procedure requires interventional radiology access, which, when coupled with the higher incidence in already resource-limited settings, may limit utility outside of large academic centers. Our patient also underwent embolization as she was deemed a poor surgical candidate during her initial presentation. While the procedure was successful in hemorrhage control, the patient still had a prolonged and arduous recovery secondary to the disease burden and other postoperative complications.

## Conclusions

Cervical cancer, unfortunately, remains a prevalent disease worldwide. Despite the focus on preventative screening and vaccinations for the prevention of cervical cancer, patients may still present with a significant cancer burden and associated complications, as demonstrated in this case. This case signifies the need for a high suspicion for possible underlying malignancy and a comprehensive exam, particularly with our patient’s presentation of recurrent UTIs and eventual significant vaginal hemorrhage in a postmenopausal female. Despite the above available interventions, our patient continued to have an arduous inpatient course, highlighting the morbidity associated with cervical cancer, even in resource-heavy environments.
